# *Portulaca oleracea* Ameliorates Diabetic Vascular Inflammation and Endothelial Dysfunction in db/db Mice

**DOI:** 10.1155/2012/741824

**Published:** 2012-03-01

**Authors:** An Sook Lee, Yun Jung Lee, So Min Lee, Jung Joo Yoon, Jin Sook Kim, Dae Gill Kang, Ho Sub Lee

**Affiliations:** ^1^College of Oriental Medicine and Professional Graduate School of Oriental Medicine, Wonkwang University, Shinyong-dong, Iksan, Jeonbuk 570-749, Republic of Korea; ^2^Hanbang Body-Fluid Research Center, Wonkwang University, Shinyong-dong, Iksan, Jeonbuk 570-749, Republic of Korea; ^3^Korea Institute of Oriental Medicine, Jeonmin-dong, Yusung-gu, Daejeon 305-811, Republic of Korea

## Abstract

Type 2 diabetes is associated with significantly accelerated rates of micro- and macrovascular complications such as diabetic vascular inflammation and endothelial dysfunction. In the present study, we investigated the protective effect of the aqueous extract of *Portulaca oleracea* L. (AP), an edible plant used as a folk medicine, on diabetic vascular complications. The db/db mice were treated with AP (300 mg/kg/day, p.o.) for 10 weeks, and AP treatment markedly lowered blood glucose, plasma triglyceride, plasma level of LDL-cholesterol, and systolic blood pressure in diabetic db/db mice. Furthermore, AP significantly increased plasma level of HDL-cholesterol and insulin level. The impairment of ACh- and SNP-induced vascular relaxation of aortic rings were ameliorated by AP treatment in diabetic db/db mice. This study also showed that overexpression of VCAM-1, ICAM-1, E-selectin, MMP-2, and ET-1 were observed in aortic tissues of untreated db/db mice, which were significantly suppressed by treatment with AP. We also found that the insulin immunoreactivity of the pancreatic islets remarkably increased in AP treated db/db mice compared with untreated db/db mice. Taken together, AP suppresses hyperglycemia and diabetic vascular inflammation, and prevents the development of diabetic endothelial dysfunction for the development of diabetes and its vascular complications.

## 1. Introduction

Macrovascular complications including atherosclerosis are the leading cause of morbidity and mortality in patients with diabetes mellitus (DM) [1, 2]. The endothelium is an early target in diabetes, and dysfunction of endothelial cells plays an important role in the diabetic vascular complications [3]. Hypertension, an impaired endothelium-dependent vasodilation, has been identified as an independent risk factor for the development of endothelial dysfunction and inflammation. Endothelin-1 (ET-1), a potent vasoconstrictor with profibrotic properties, is chronically elevated in diabetes. Recent study indicated that an increased endothelin-1 level of plasma is involved in diabetic complications [4]. In cardiovascular disease, the matrix metalloproteinases (MMPs) can result in a tissue injury and inflammation. An increased MMP activity contributes to cardiac and vascular complications in experimental hypertension and atherosclerosis models [5, 6].

Inflammatory cytokines and adhesion molecules seem to play main roles in the pathogenesis of atherosclerosis and diabetic vascular complications [7]. Adhesion of monocyte and endothelial cell is an important early event in vascular inflammation [8, 9]. In the process of inflammation, monocytes are recruited to sites of endothelial cell injury and roll along the vascular endothelium, where they become activated by surface-bound chemokines. The monocytes adhere strongly to the vascular endothelium and transfer through the endothelial cells monolayer [10, 11]. Molecules such as endothelial vascular cell adhesion molecule-1 (VCAM-1), intercellular adhesion molecule-1 (ICAM-1), and E-selectin mediate the adhesion of monocytes [12]. db/db mice have a point mutation in the leptin receptor gene and are a well-established model of type 2 diabetes. The mice are obese, hyperglycemic, and insulin resistant at early age and progress to more severe diabetes, resembling type 1 diabetes with a pancreatic beta cell dysfunction after 16 weeks of age [13, 14].

*Portulaca oleracea *L. (Portulacaceae) is an edible plant and has been used as a folk medicine in many countries, acting as a diuretic, febrifuge, antiseptic, antispasmodic, and vermifuge [15, 16]. It has been shown to play pharmacological roles, including antibacterial [17], analgesic [18], skeletal muscle-relaxant [19], and wound-healing [20] activities. Many studies have also shown that the major bioactive components of *Portulaca oleracea* are flavonoids, coumarins, monoterpene glycoside, and alkaloids [21, 22]. Some research results indicated that *Portulaca oleracea *could also be used to reduce the incidence of cardiovascular diseases [23]. Thus, the purpose of this study was to examine the protective effect of an aqueous extract of *Portulaca oleracea* (AP) against hyperglycemia, diabetic metabolic disorder, and diabetic vascular complications in diabetic db/db mice.

## 2. Materials and Methods

### 2.1. Extraction of *Portulaca oleracea*

The *Portulaca oleracea* was purchased from the Herbal Medicine Co-operative Association in Jeonbuk Province, Korea. Herbarium voucher specimen (No. HBE121) was deposited in the herbarium of the Professional Graduate School of Oriental Medicine, Wonkwang University, Iksan, Jeonbuk, South Korea. Dried aerial parts of *P. oleracea* (400 g) was extracted with 5.5 L of boiled distilled water at 100°C for 2 h. The aqueous extract was centrifuged at 1,000 g for 20 min at 4°C and supernatant was filtered with Whatman No. 3 filter paper, and then concentrated using rotary evaporator. The supernatant extract was lyophilized to produce a powder, which was then kept at 4°C until using this experiment. The yield of the water extract of *P. oleracea* was approximately 22.8% of plant powder.

### 2.2. Experimental Animals

Male C57BL/KsJ-db/db mice and wild-type C57BL/6J mice were obtained from the Central Lab. Animal Inc. (Seoul, Korea) at age 6 weeks. At 8 weeks of age, fasting blood glucose was determined in each mice, and then divided into four groups with 10 mice; wild type mice, db/db mice, db/db mice treated with AP (300 mg/kg/day, p.o.) and db/db mice treated with rosiglitazone by sonde (Alexis Biochemicals, San Diego, CA, USA; 10 mg/kg/day, p.o. final volume 0.3 mL). The peroxisome proliferator-activated receptor-*γ* (PPAR-*γ*) agonist, rosiglitazone was chosen as a positive control, which is an anti-diabetic agent for the treatment of type 2 diabetes. All mice fed the purified AIN-76A rodent diet (Dyets, Inc., Bethlehem, PA, USA) for 10 weeks. All mice were maintained under standard light (12 h light/dark), temperature (22 ± 2°C) and humidity (40 ± 10%) condition. All procedures were approved by the Institutional Animal Care and Utilization Committee for Medical Science of Wonkwang University.

### 2.3. Plasma Biochemical Analysis

The blood glucose concentration was measured with the whole blood obtained from the tail veins after withholding food for 6 h using a Onetouch Ultra Blood Glucose Meter and Test Strip (LifeScan Inc., CA, USA). Blood samples were taken by periorbital vein for biochemical analysis. Plasma insulin levels were measured based on ELISA method using commercial mice insulin ELISA kit (Shibyagi Co., Gunma, Japan). The total cholesterol, HDL cholesterol, LDL cholesterol, and triglyceride levels in plasma were measured enzymatically using commercially available kits (Wako Pure Chemical Industries, Osaka, Japan).

### 2.4. Measurement of Systolic Blood Pressure

Systolic blood pressure (SBP) and heart rate were determined while mice were conscious using a noninvasive tail-cuff monitor (MK2000; Muromachi Kikai, Tokyo, Japan). Values are presented as the mean ± SEM of five measurements.

### 2.5. Preparation of Aortic Rings

The mice aortae were carefully dissected and placed into ice-cold Kreb's solution (pH 7.4) containing 118 mM NaCl, 4.7 mM KCl, 1.1 mM MgSO_4_, 1.2 mM KH_2_PO_4_, 1.5 mM CaCl_2_, 25 mM NaHCO_3_, and 10 mM glucose. The aortae were removed free of connective tissue and fat and then cut into rings with a width of approximately 2-3 mm. All dissecting procedures were carried out carefully to protect the endothelium from unintentional damage. Endothelial relaxant response was measured by responding ACh and SNP on phenylephrine-(1 *μ*M)-induced aortic rings [24].

### 2.6. Recording of Isometric Vascular Tone

The aortic rings were suspended, by means of two L-shaped stainless-steel wires inserted into the lumen, in a tissue bath containing Kreb's solution (pH 7.4) at 37°C. 

Mixed gas contained 95% O_2_, and 5% CO_2_ was continuously bubbled through the bath. The baseline load placed on the aortic rings was 1.0 g. Changes in isometric tension were recorded using a force displacement transducer (Grass FT 03, Quincy, MA) connected to a Grass polygraph recording system (Model 7E). The aortic rings were washed every 10 min with Kreb's solution until the tension returned to the basal level. After stabilized, the aortic rings were contracted with phenylephrine (1 *μ*M) to obtain a maximal response, and then a concentration-dependent response curve to acetylcholine (Ach) and sodium nitroprusside (SNP) was determined in thoracic aorta, respectively.

### 2.7. Western Blot Analysis

Aortae were homogenized with a buffer containing 250 mM sucrose, 10 mM triethanolamine, 10 mM acetic acid, 1 mM EDTA, 1 mM phenylmethylsulfonyl fluoride (PMSF), 1 mM benzamidine hydro-chloride hydrate, and 1 mM dithiothreitol, at pH 7.4. The homogenates were then centrifuged at 8,000 rpm for 10 min at 4°C, and the supernatant was centrifuged at 13,000 rpm for 5 min at 4°C. The proteins were separated by 10% sodium dodecyl sulfate-polyacrylamide gel electrophoresis (SDS-PAGE) and transferred to Hybond-ECL nitrocellulose membranes (Amersham, Arlington Heights, IL). Membranes were blocked with 5% nonfat milk for 1 h and then probed primary antibodies with ICAM-1, VCAM-1, E-selectin, and MMP-2 (1 : 1000; Santa Cruz Biotechnology, CA) for overnight at 4°C. After washing with TBST, they were probed with horseradish peroxidase-conjugated secondary antibody. The immunoreactive bands were visualized using ECL kit (Upstate Biotechnology, Lake Placid, NY) according to the manufacturer's instructions and quantified using a ChemiDoc image analyzer (Bio-Rad, Hercules, CA).

### 2.8. Immunoflorescence Staining of Insulin in Pancreatic Islets

Slides of the pancreatic frozen section were incubated with 10% nonimmune goat serum for 1 h at room temperature to block nonspecific staining, and incubated with a primary antibodies of insulin (1 : 100, Santa Cruz Biotechnology) for overnight at 4°C. After washing, fluorescein-conjugated goat anti-rabbit IgG and Alexa Fluor 594-conjugated donkey anti-goat IgG (1 : 200, Molecular Probes, Carlsbad, CA) were incubated for 1 h at room temperature. After washing at 3 times, the sections were mounted and observed by Olympus fluorescence microscopy. The expressions of insulin in pancreas were observed by Olympus microscopy equipped with an Olympus DP 70 camera. For the quantitative analysis, the average score of 10–20 randomly selected area was calculated using NIH Image analysis software, Image J (NIH, Bethesda, MD).

### 2.9. Immunohistochemical Staining of VCAM-1 and ET-1 in Aortic Tissue

Isolated aortic tissues were fixed by immersion in 4% paraformaldehyde for 48 h at 4°C and incubated with 30% sucrose for 2 days. Each aorta was embedded in OCT compound (Tissue-Tek, Sakura Finetek, Torrance, CA), frozen in liquid nitrogen, and stored at −70°C. Frozen sections, 10 *μ*m thick, were cut with a Shandon Cryotome SME (Thermo Electron Corporation, Pittsburg, PA) and placed on poly-L-lysine-coated slides. The slides were air dried overnight at room temperature, wrapped, and stored at −70°C until immunostaining. Slides were immunostained by Invitrogen's Histostain-SP kits using the Labeled-Strept-avidin-Biotin (LAB-SA) method. Slides were immersed in 3% hydrogen peroxide for 1 min at room temperature to block endogenous peroxidase activity and rinsed with PBS. And then, slides were incubated with 10% nonimmune goat serum for 20 min at room temperature to block nonspecific staining and incubated with a primary antibodies of VCAM-1 (1 : 500; Chemicon International Inc., Temecula, CA), ET-1 (1 : 500; Oncogene, CA) in humidified chambers for overnight at 4°C. All slides were incubated with biotinylated secondary antibody for 20 min at room temperature and then incubated with horseradish peroxidase-conjugated streptavidin for 20 min at room temperature, followed by detection with 3-amino-9-ethylcarbazole (AEC) as chromogen and counterstaining with hematoxylin (Zymed, CA). For the quantitative analysis, the average score of 10–20 randomly selected area was calculated using NIH Image analysis software, Image J (NIH, Bethesda, MD).

### 2.10. Statistical Analysis

Values are shown as mean ± SE. Statistical analyses were performed using analysis of variance followed by the Student's *t-*test and one-way ANOVA. Differences with a value of *P* < 0.05 were considered statistically significant.

## 3. Results

### 3.1. Effect of AP on Blood Glucose Level

**Figure 1:**
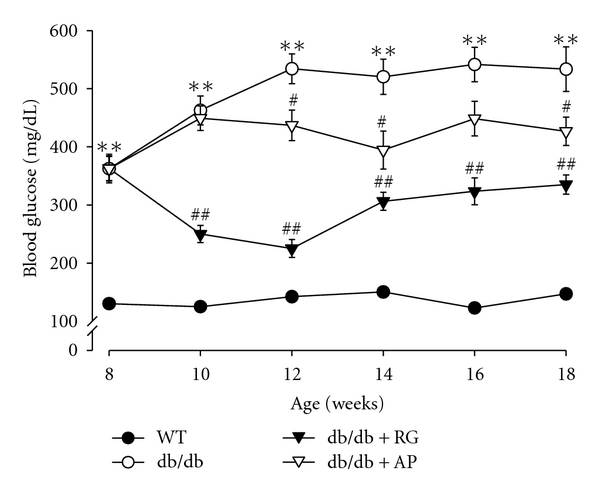
Effect of AP on weekly blood glucose levels in db/db mice. Values are expressed as mean ± SE (*n* = 10); **P < 0.01 versus WT; ^#^ *P* < 0.05, ^##^ *P* < 0.01 versus db/db control.

The blood glucose level of untreated db/db mice was significantly higher than that of the wild-type mice during entire experimental period. Interestingly, the blood glucose levels were markedly reduced in AP-treated db/db mice compared to the untreated db/db mice over the 12 weeks of age. Similarly, these levels of RG-treated db/db mice were significantly decreased over the 10 weeks of age (Figure 1). No mortality was observed, and AP was found to be safe at given doses.

### 3.2. Effect of AP on Systolic Blood Pressure

**Figure 2:**
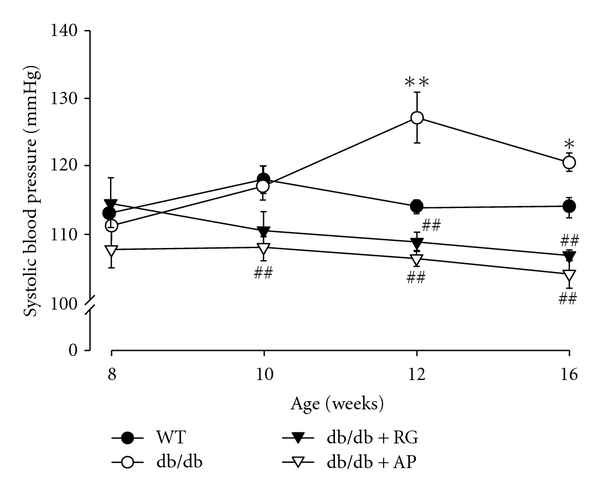
Effect of AP on weekly systolic blood pressure levels in db/db mice. Values are expressed as mean ± SE (*n* = 10); *P < 0.05, **P < 0.01 versus WT; ^##^ *P* < 0.01 versus db/db control.

The systolic blood pressure (SBP) level after 4 weeks of treatment significantly increased in untreated db/db mice. The AP-treated db/db mice showed a significant decrease in the SBP level compared with the untreated db/db mice during all the experimental period. As similar in level, the RG-treated db/db mice indicated a remarkable decrease in that level compared to the untreated db/db mice (Figure 2).

### 3.3. Effect of AP on Vascular Tension

**Figure 3:**
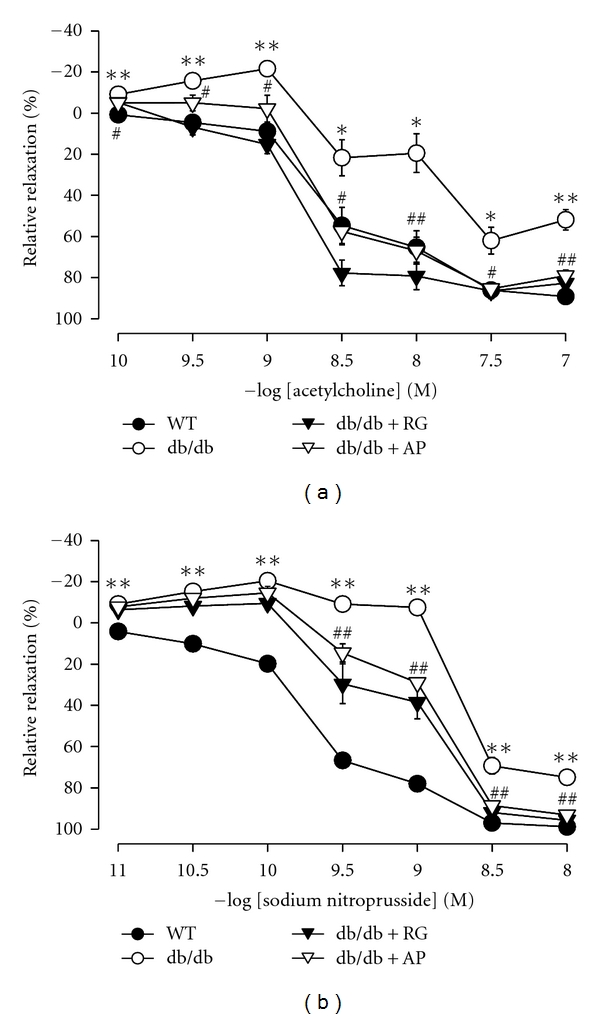
Effect of AP on the ACh-induced (a) and SNP-induced (b) relaxation of thoracic aorta from db/db mice. Values are expressed as mean ± SE (*n* = 5); *P < 0.05, **P < 0.01 versus WT; ^#^ *P* < 0.05, ^##^ *P* < 0.01 versus db/db control.

Vascular responses to ACh (1 × 10^−10^ to 1 × 10^−7^ M) and SNP (1 × 10^−11^ to 1 × 10^−8^ M) were measured in thoracic aorta. Endothelium-dependent relaxant responses to Ach were significantly impaired in the untreated db/db mice compared to wild-type mice. The impairment of vasorelaxation was markedly ameliorated by treatment with AP (Figure 3(a)). Similarly, smooth muscle dilatory responses to SNP markedly decreased in the untreated db/db mice compared to wild-type mice, which were significantly recovered by treatment with AP (Figure 3(b)).

### 3.4. Effect of AP on Plasma Biomarker

**Figure 4:**
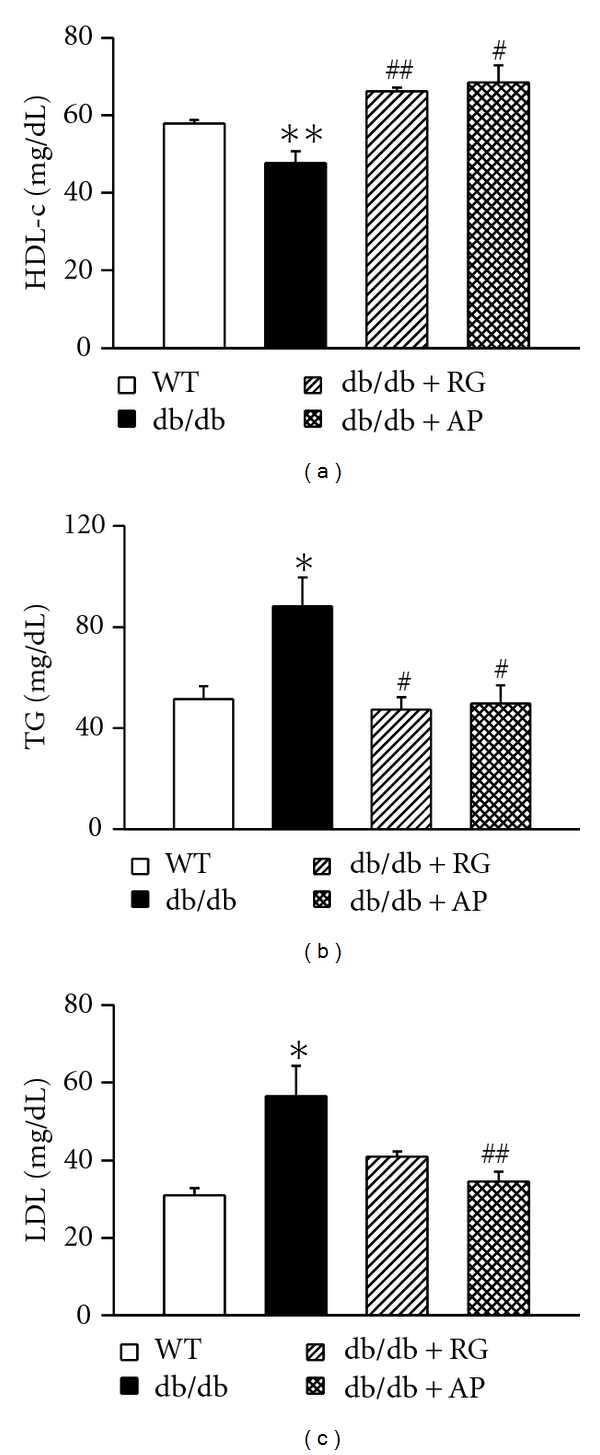
Effect of AP on HDL cholesterol (a), triglyceride (b), and LDL cholesterol (c) levels of plasma in db/db mice. Values are expressed as mean ± SE (*n* = 5); *P < 0.05, **P < 0.01 versus WT; ^#^ *P* < 0.05, ^##^ *P* < 0.01 versus db/db control.

The plasma insulin level was measured at the end of the study. Our results showed that the plasma insulin level of the AP-treated db/db mice was significantly higher than that of the untreated db/db mice. Therefore, treatment with AP markedly inhibited the age-related decrease of plasma insulin level in db/db mice. The plasma insulin level of the RG-treated db/db mice was significantly higher than that of the untreated db/db mice (data not shown). The plasma triglyceride and low-density lipoprotein (LDL) cholesterol levels of the untreated db/db mice significantly increased compared with wild-type mice. The treatment with AP significantly lowered these levels in the db/db mice (Figures 4(b) and 4(c)). In contrast, treatment with AP markedly increased the high-density lipoprotein (HDL) cholesterol in the db/db mice (Figure 4(a)).

### 3.5. Effect of AP on Vascular Inflammation in Aortic Tissue

**Figure 5:**
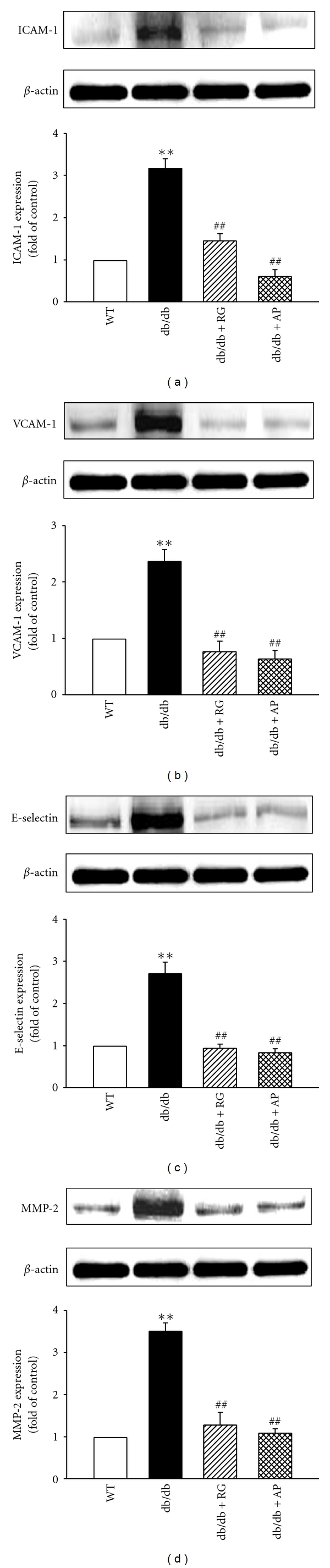
Effect of AP on ICAM-1 (a), VCAM-1 (b), E-selectin (c), and MMP-2 (d) expression in the aorta of db/db mice. Western blots and corresponding densitometric analyses of ICAM-1, VCAM-1, E-selectin, and MMP-2 in aortic tissue. Values are expressed as mean ± SE (*n* = 3); **P < 0.01 versus WT; ^##^ *P* < 0.01 versus db/db control.

Vascular adhesion molecules, ICAM-1 (Figure 5(a)), VCAM-1 (Figure 5(b)), and E-selectin (Figure 5(c)) were evaluated quantitatively in aortic tissue by Western blot analysis. Expressions of ICAM-1, VCAM-1, and E-selectin in untreated db/db mice significantly increased compared with wild-type mice, respectively. However, treatment with AP effectively reduced the expressions of ICAM-1, VCAM-1, and E-selectin in db/db mice. Matrix metalloproteinase- (MMP-) 2 was also measured using Western blot in aortic tissue. MMP-2 induced degradation of proteins in the extracellular matrix. In untreated db/db mice, expression of MMP-2 markedly increased compared with wild-type mice. AP treatment significantly lowered this overexpression level in db/db mice (Figure 5(d)).

**Figure 6:**
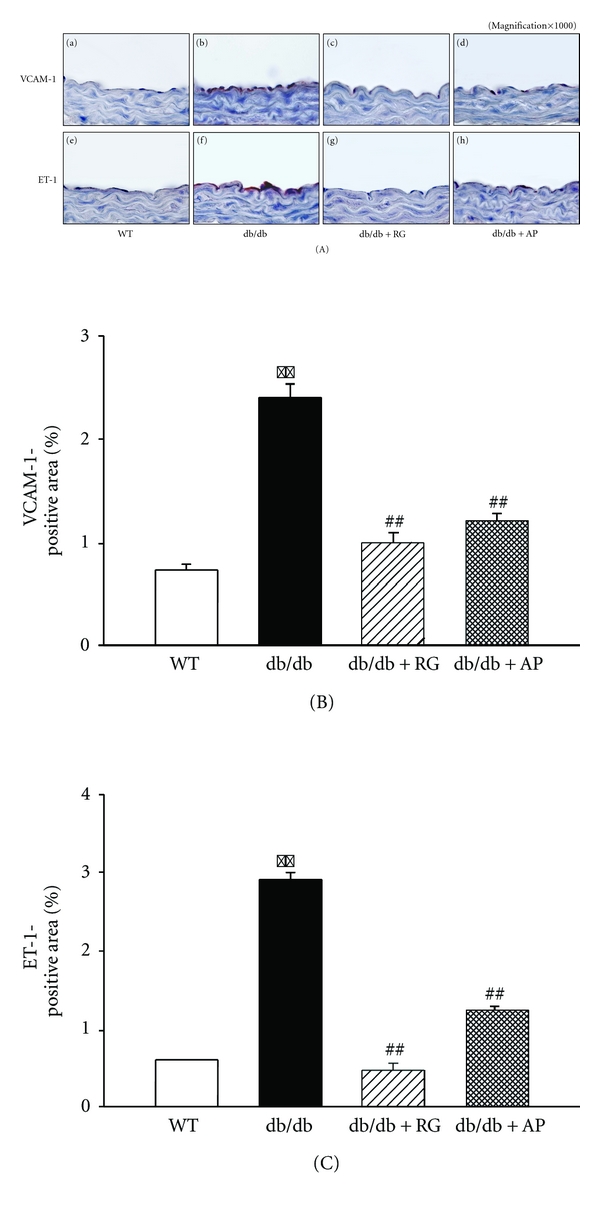
Effect of AP on VCAM-1 and ET-1 immunoreactivity in the aorta of db/db mice. (A) Immunohistochemical staining of VCAM-1 or ET-1 in aorta from (a, e) WT, (b, f) untreated db/db mice, (e, g) db/db mice treated with rosiglitazone (10 mg/kg/day), and (d, h) db/db mice treated with AP (300 mg/kg/day). Lower panels show quantitative analysis of VCAM-1- (B) and ET-1- (C) -positive area. The average score of 5–10 randomly selected sites per section of aorta was calculated. Data expressed as mean ± SE; **P < 0.01 versus WT; ^##^ *P* < 0.01 versus db/db control. Original magnification:1000x.

Immunohistochemical staining showed that VCAM-1 and ET-1 were overexpressed in the aortic endothelial layers of untreated db/db mice compared with wild-type mice, which were markedly reduced by treatment with AP (Figure 6).

### 3.6. Effect of AP on Immunoreactivity for Insulin in Pancreatic Islets

**Figure 7:**
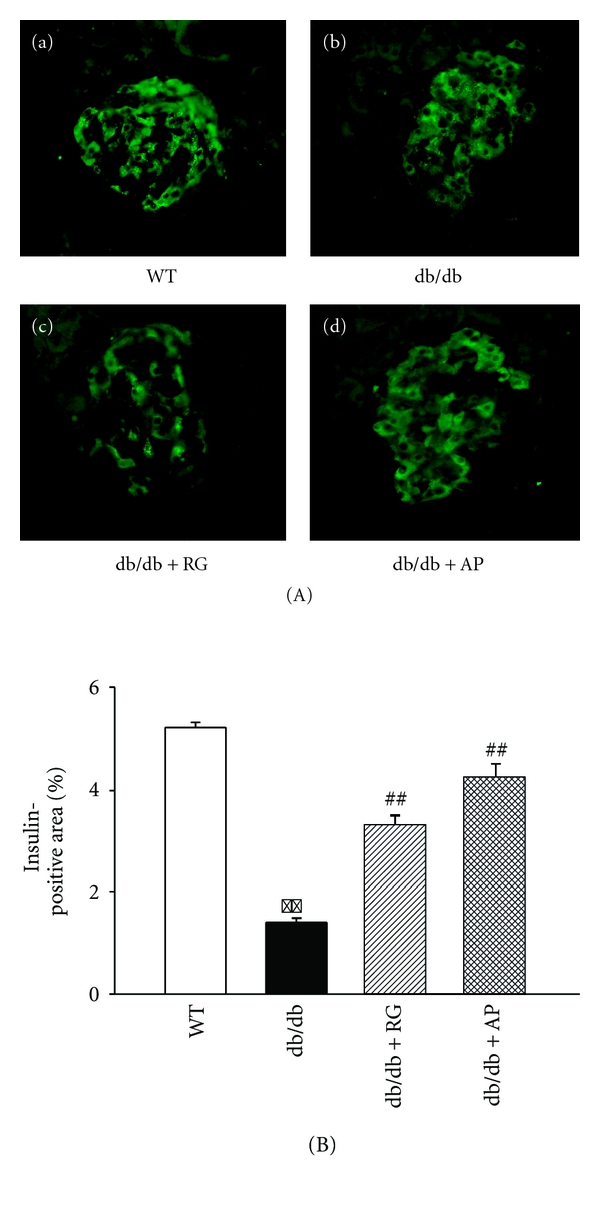
Immunofluorescence staining of insulin in the pancreas. Representative histological sections of pancreatic islets of (a) WT, (b) untreated db/db mice, (c) db/db mice treated with rosiglitazone (10 mg/kg/day), and (d) db/db mice treated with AP (300 mg/kg/day) incubated with anti-insulin antibodies. (B) Quantitative analysis of insulin-positive area. Data expressed as mean ± SE (*n* = 5); **P < 0.01 versus WT; ^##^ *P* < 0.01 versus db/db control. Original magnification:1000x.

In untreated db/db mice, immunofluorescent staining of the pancreatic tissues represented weak insulin immunoreactivity in the islets of Langerhans. In AP-treated db/db mice, there was an indicated strong insulin immunoreactivity in the islets of Langerhans (Figure 7).

## 4. Discussion

In the present study, we clearly demonstrate that the aqueous extract of *Portulaca oleracea *(AP) has an antidiabetic effect by prevention of the developed diabetic symptoms and suppression of diabetic vascular complications in type 2 diabetic db/db mice. Diabetes is associated with significantly accelerated rates of cardiovascular complications such as atherosclerosis and hypertension. Recent studies indicate that diabetic atherosclerosis is not simply a disease of hyperlipidemia but also an inflammatory disorder [1, 25]. Large clinical trials in both type 1 and type 2 diabetes have also showed that hyperglycemia plays an important role in the pathogenesis of vascular complications. In addition, type 2 diabetes occurs in the cluster of cardiovascular risk factors, hypertension, high triglyceride levels, low HDL-cholesterol levels, increased LDL-cholesterol levels, hyperinsulinemia, insulin resistance, and chronic inflammation, all of which accelerate diabetic vascular complications [26]. All of these factors induce a state of constant and progressive damage to the vascular wall, indicated by inflammatory process and endothelial dysfunction [27]. Accordingly, endothelial dysfunction and inflammation in Type 2 diabetes are progressive and closely interrelated [28]. Endothelial dysfunction was initially identified as impaired vasodilation to specific stimuli such as ACh or bradykinin; therefore, improvement of endothelial function is predicted to regulate lipid homeostasis [29]. Our results showed that AP treatment markedly reduced blood glucose, triglyceride, LDL cholesterol, and systolic blood pressure levels in diabetic db/db mice. Furthermore, treatment with AP significantly increased the level of HDL cholesterol in diabetic db/db mice. The endothelium can sense changes or abnormalities in blood flows and pressures, and vascular endothelium exists between circulating blood and vascular smooth muscle is playing the important role in modulation of vascular tone [30]. Impaired relaxation of aorta was induced by ACh in obese diabetic fatty rats as a consequence of endothelial dysfunction [28, 31]. In our data, the impairment of acetylcholine- (ACh-) and sodium-nitroprusside- (SNP-) induced vascular relaxation of aortic rings in diabetic db/db mice was ameliorated by treatment with AP. Vasoconstrictor endothelin (ET)-1 expression was significantly increased in the aorta in db/db mice; however, treatment with AP markedly decreased the levels of ET-1. In addition, impaired acetylcholine-dependent vasodilation was recently associated with increased levels of the plasma soluble E-selectin and P-selectin in essential hypertension. COX-2 upregulation and vascular smooth muscle contractile hyperreactivity were reported in spontaneous diabetic db/db mice [32]. We showed the overexpression of VCAM-1, intercellular cell adhesion molecule- (ICAM-) 1, E-selectin, and MMP-2 in aortic tissue of untreated db/db mice. Interestingly, treatment with AP significantly suppressed these expressions. It suggested that diabetic endothelial dysfunction was related with vascular inflammation, and AP treatment recovered it. These findings, at least in part, indicate that AP may protect against the initiation and development of diabetic atherosclerosis by improving lipid homeostasis.

Plasma insulin levels of db/db mice are known to be age dependent. However, when the db/db mice reach at 12 to 24 weeks old, islet develops *β*-cell necrosis, hyperinsulinemia is diminished, and the mice manifest symptoms of insulin deficiency [33]. Type 2 diabetes is characterized by pancreatic *β*-cell dysfunction. Normal pancreatic *β* cells can compensate for the insulin resistance by increasing insulin secretion. However, extensive exposure of pancreatic *β* cells induced by high glucose levels induces *β*-cell dysfunction that is associated with reduced insulin secretion [34]. In the present study, we observed that plasma insulin concentration was significantly higher in the AP-treated db/db mice compared to the untreated db/db mice. Therefore, these data suggest that the plasma insulin level in the db/db mice may be gradually declined, whereas AP may inhibit the age-dependent insulin reduction by a reduction of *β*-cell mass. Moreover, in untreated db/db mice, immunostaining of the pancreatic tissues represented weak insulin secretion in the islets of Langerhans. However, there was an indicated strong insulin secretion in the islets of Langerhans by treatment with AP. A previous study demonstrated that* P. oleracea* extract exhibited potent antioxidant property in streptozotocin diabetic rats. However, it showed only moderate antidiabetic activity [35]. In addition, AP not ethanolic extract significantly inhibited DNA damage in human lymphocytes [36]. The present study demonstrated that AP exhibited hypoglycemic action more than rosiglitazone in db/db mice. These results suggested that vascular protective role of AP may be involved in type 2 diabetes. Further study is required to clarify the mechanism of AP in db/db mice.

Taken together, aerial parts of *P. oleracea* treatment markedly ameliorated hyperglycemia and impairment of insulin secretion and prevented diabetic endothelial dysfunction and vascular inflammation in type 2 diabetic db/db mice. In conclusion, AP has an antihyperglycemic effect and preventive effect on pathological mechanism of diabetic vascular complications. These findings support the palliative effect of AP in the development of diabetes and its vascular complications.
